# Malnutrition: Modulator of Immune Responses in Tuberculosis

**DOI:** 10.3389/fimmu.2017.01316

**Published:** 2017-10-18

**Authors:** Padmapriyadarsini Chandrasekaran, Natarajan Saravanan, Ramalingam Bethunaickan, Srikanth Tripathy

**Affiliations:** ^1^Department of Clinical Research, National Institute for Research in Tuberculosis, Chennai, India; ^2^Department of Biochemistry and Clinical Pharmacology, National Institute for Research in Tuberculosis, Chennai, India; ^3^Department of Immunology, National Institute for Research in Tuberculosis, Chennai, India; ^4^National Institute for Research in Tuberculosis, Chennai, India

**Keywords:** malnutrition and tuberculosis, nutrition and immunity of tuberculosis, vitamin D, sphingolipid, food supplementation for tuberculosis, *Mycobacterium tuberculosis*

## Abstract

Nutrition plays a major role in the management of both acute and chronic diseases, in terms of body’s response to the pathogenic organism. An array of nutrients like macro- and micro-nutrients, vitamins, etc., are associated with boosting the host’s immune responses against intracellular pathogens including *mycobacterium tuberculosis* (*M.tb*). These nutrients have an immunomodulatory effects in controlling the infection and inflammation process and nutritional deficiency of any form, i.e., malnutrition may lead to nutritionally acquired immunodeficiency syndrome, which greatly increases an individual’s susceptibility to progression of infection to disease. This narrative review looks at the various mechanisms by which nutrition or its deficiency leads to impaired cell mediated and humoral immune responses, which in turn affects the ability of an individual to fight *M.tb* infection or disease. There is very little evidence in the literature that any specific food on its own or a specific quantity can alter the course of TB disease or be effective in the treatment of malnutrition. Further clinical trials or studies will be needed to recommend and to better understand the link between malnutrition, tuberculosis, and impaired immunity.

## Introduction

Tuberculosis (TB) has existed for millennia and continues to remain a major global public health problem. It is an infectious disease caused by *Mycobacterium tuberculosis* (*M.tb*), typically affects the lungs but can also affect other sites and spreads when a person with TB expels the bacteria into the air while coughing or sneezing. TB is one of the top 10 causes of death worldwide and the leading cause of death from an infectious disease (1). World Health Organization estimates that, in 2016, there were 10.4 million new TB cases (including 1.2 million among HIV-infected individuals) worldwide. 90% of cases were in adults and 10% in children with a male:female ratio of 1.6:1. There were 1.4 million TB deaths in 2015, with an additional 0.4 million deaths resulting from TB disease among HIV-infected individuals (1). 5–15% of the estimated two to three billion people infected with *M.tb* will develop TB disease during their lifetime, the probability increasing among people with HIV (1).

### Pathogenesis of TB

Pathogenesis of TB involves various steps beginning from the exposure to *M.tb*, the development of infection, the progression of infection to disease, and finally the outcome of the disease. A complex interaction between diverse groups of cells and cytokines are involved in the progression of one stage to the next in the journey of TB disease.

#### Exposure to *M.tb*

Exposure to *M.tb* occurs when an active TB patient releases the infectious droplets in the air during coughing, sneezing, or talking. The bacteria in the droplet nuclei are infective to another person when inhaled.

#### Primary Infection

The *M.tb* in the droplet nuclei when inhaled by another individual is carried to the alveoli where they infect macrophages and multiply inside them. Within 2–6 weeks, the infection is followed by cellular immune response generated by CD4+ T-lymphocytes and infected macrophages release cytokines and chemokines. In 70–90% of instances, the immune response of an individual is strong enough, and it will fight off the TB bacteria and not become infected.

#### Latent TB Infection (LTBI)

During *M.tb* infection, the host immune response is able to contain the mycobacterial infection at the site of infection in approximately 30% of cases, but is unable to “sterilize” them. These foci later become associated with a state of LTBI with *M.tb* where persons are healthy, asymptomatic, and the infection is present in an enclosed environment in a non-transmissible state. The cellular immune response leads to the formation of a granuloma and the infection is curtailed. It also generates a delayed-type hypersensitivity reaction. Globally in 2014, approximately 1.7 billion individuals were estimated to be latently infected with *M.tb* and form a reservoir for future TB disease (2).

#### Progression from LTBI to Active TB Disease

This can occur with weakening of immune responses. If person is unable to control the initial infection, it can progress to active primary disease as in children. If the infection is curtailed and goes into latency, with the weakening of the immune system, it can progress to disease even months or years later; by breakdown of granuloma and active uncontrolled replication of mycobacteria with resultant disease in lungs and other organs (3).

The likelihood of getting infected with *M.tb* as well as the subsequent development of active disease depends upon number of factors like the infectivity of the source case, proximity and duration of contact, susceptibility of the host and various social, behavioral, economic, and environmental factors like undernutrition, overcrowding, indoor air pollution, smoking, and alcohol addiction (4–7). Of these, undernutrition is the single most important predisposing factor for TB in many resource limited settings (8).

### Nutrition and TB

An array of nutrients like macro- and micro-nutrients (vitamins, minerals, and trace elements) are associated with boosting the immune responses against intracellular pathogens like *M.tb*. These nutrients have an immunomodulatory effect in controlling the infection and inflammation process. Protein-energy or micronutrient deficiency leads to altered immune-homeostasis, which greatly increases an individual’s susceptibility to infections or progression of infection to disease. Human immunodeficiency virus is the most well-known cause of immunodeficiency worldwide leading on to acquired immunodeficiency syndrome, but the most prevalent cause of immunodeficiency is malnutrition otherwise known as nutritionally acquired immunodeficiency syndrome. Though studies have shown that both macro- and micro-nutrient deficiency increases the risk of TB by affecting host immunity (9), evidences to show the exact mechanism of how nutrition affects the host immune response is still not clear.

### Immunity in TB

*Mycobacterium tuberculosis* infection induces both cell-mediated and humoral immune responses in an individual. It has been shown that B-cell deficiency leads to a higher bacterial burden and worse outcome following *M.tb* infection while antibodies to *M.tb* enhance internalization of mycobacteria by phagocytic cells (10, 11). These antibodies significantly increase the ability of macrophages to kill intracellular mycobacteria and lead to marked increase in *M.tb* specific cell-mediated immunity (11). Various types of inflammatory cytokines of both innate and adaptive immune systems coordinate the immune response of an individual to *M. tb* infection (12).

### Malnutrition and Immunology

Malnutrition affects both the innate and adaptive immunity of an individual rendering them susceptible to a variety of infections. Phagocytosis and complement cascade are two main mechanisms involved with elimination of pathogenic organisms from the body. Complement system by itself can destroy microorganisms or the complement receptors present on the surface of phagocytes can mediate capture of pathogens. With malnutrition, both functions get compromised—the opsonic complement factor C3 as well as the phagocytic ability to ingest and kill pathogens are also reduced considerably (10, 11). In addition, the functioning of various antigen-presenting cell types like the B lymphocytes, macrophages, dendritic cells (DCs), and Kupffer cells are decreased in malnutrition (13). We have tried to give a brief narrative review of the role of different nutrients in altering the inflammatory processes and, thereby, host immunity to diseases like TB in the following section.

#### Protein Energy Malnutrition (PEM)

Malnutrition is known to have direct effects on T cells. Severe PEM provokes atrophy of thymus as well as peripheral lymphoid organs, which in turn reduces cell number (leukopenia), decreases CD4/CD8 ratio, increases numbers of CD4 and CD8 double-negative T cells, and increases the number of immature T cells in the peripheral blood (14). A major decrease in the expression of CD25 and CD27 (the molecules required for T cell activation and proliferation) was also demonstrated. Immune response of the gut mucosa is also affected by malnutrition. It results in flattened hypotrophic microvilli, reduced IgA secretion, and lymphocyte counts in Peyer’s patches (15). Malnourished children have shown reduced production of type 1 cytokines (IL-2 and IFN-γ), which are the main mediators of immunity (16). Such changes in cell-mediated immunity lead to increased susceptibility of an individual to infection. Similarly, low serum albumin levels (<2.7 g/dl) has been shown to be strongly and independently associated with in-hospital deaths due to TB (aOR 3.38, 95% CI 1.51–7.59; *P* = 0.001) (17).

#### Essential Fatty Acids

There are two classes of essential fatty acids [n6 and n3 polyunsaturated fatty acids (n6 and n3PUFA)], which share the common enzyme system for their metabolism and their metabolic end products are mostly distinct in action. There are contrasting evidences to support the effects of these fatty acids *in M.tb* survival and proliferation (18). One of the important mechanisms by which the *M.tb* survives in the macrophages is by inhibiting the membrane actin assembly, which is essential for the maturation of phagosomes. The *in vitro* studies on effect of lipids on *M.tb* survival in macrophages showed that adding eicosapentaenoic acid (n3 PUFA) to culture medium favor *M.tb* growth. The suggested mechanism for this is inhibition of actin filament assembly while arachidonic acid (n6 PUFA) promotes it (19). The same group tested the *in vitro* results in an animal model and found a contrasting observation that n3 PUFA promoted *M.tb* clearance while n6PUFA helped *M.tb* survival suggesting the presence of complex metabolic and immunologic interplay (20). The enrichment of anti-inflammatory n3PUFA in host cells is also beneficial for T-cell mediated immune reactions but detrimental to macrophage-mediated microbial clearance (21). The *M.tb* survival in the host depends on the regulation of “Eicosanoids”—the complex metabolic end products of n6PUFA. The virulent *M.tb* diverts the metabolic pathway toward the production of lipoxin A4 and thereby inhibits cyclooxygenase 2 and decreases the levels of prostaglandin E2 (PGE2), which is a metabolic end product of arachidonic acid. By limiting the PGE2, which is involved in cellular repair, *M.tb* produces a necrotic environment in macrophages and thereby proliferates further.

#### Sphingolipids

Sphingolipids are structural lipids, which include *sphingomyelin, ceramide, and sphingosine 1-phosphate*, which are abundant in neuronal tissue and considered important bioactive molecule of inflammation. Sphingosine-1-phosphate, with effects over calcium regulation, expression of growth factors and inflammatory cytokines are of major interest in the regulation of *M.tb* survival (22). There are several mechanisms proposed for the survival and proliferation of *M.tb* in the host cell by which the *M.tb* evades the defense mechanism of the host. One of them involves direct action over the bioactive lipids and thereby altering the calcium influx in to the cytosol. Upon binding of microbes with the surface membrane receptors of the macrophage, an enzyme called sphingosine kinase 1 (SPK1) gets activated. The activated SPK1 phosphorylates the sphingosine to sphingosine 1 P (S1P) and the bioactive S1P initiates a cascade of events to release the Ca^2+^ in the cytosol, which is essential for the maturation of the phagolysosome. The *M.tb* cell wall glycolipid lipoarabinomannan (LAM) dephosphorylates the SPK1 by stimulating the Src homology region 2 domain-containing phosphatase1 (SHP1) in host monocytes and prevents the translocation of SPK1 to the membrane and thereby prevents the maturation of the phagosome. The inhibitory activity of LAM over SPK1 is also very effective in decreasing the inflammatory molecules such as TNF-α, IL-12, etc., of the host (23). The immunomodulatory actions of *M.tb* such as intracellular release of LAM help *M.tb* to evade the adaptive immune clearance by suppression of the maturation of phagolysosome.

#### Vitamins

Many studies have reported vitamins as a key mediator of innate immune system by regulating the functions of both macrophages and DCs and immunomodulating the process of antibacterial functions, autophagosomes formation, autophagy, and cytokine production. Multiple vitamins have been shown to have a role in immunity against *M.tb* infection or disease (24–28).

Vitamin D has been recognized as a vital modulator of both innate and adaptive immune response against TB infection, enhancing the antimicrobial properties of the phagocytes viz., monocytes, macrophages, DCs, and neutrophils.

#### Modulator of Innate Immune Response

Essentially, 1, 25-dihydroxyvitamin D3 induces the differentiation of monocytes into macrophages at the site of infection and enhance the uptake of the bacterium by promoting phagocytosis (29, 30); upregulate specific markers like CD14, mannose receptor, DC sign over the surface of antigen-presenting cells to boost the phagocytic activity, and intracellular killing (30). Besides, Vitamin D also induces the expression of several antimicrobial peptides, particularly cathelicidin (*CAMP*) and β-defensin 2(*DEFB4*). Vitamin D also induces hCAP18, which triggers the autophagy mechanism of the infected cells, by mediating phagosome–lysosome fusion and plays a vital role in the elimination of the intracellular *M.tb* (31). In addition, vitamin D is found to downregulate the expression of HAMP (hepcidin antibacterial protein), which aids in the intracellular transport of iron, thereby suppressing the growth of the bacteria within macrophages (32).

#### Modulator of Adaptive Immune Response

Besides macrophages, DCs (especially the myeloid DCs) also get modulated during the antigen presentation process, by Vitamin D, during mycobacterial infection. Vitamin D suppresses DC differentiation and maturation by downregulating NF-Kb through transcription control of *relb* gene (33). Vitamin D also modulates T helper cells by downregulating the production of proinflammatory cytokines such as interferon-gamma (IFN-γ), interleukin (IL-6, IL-12), tumor necrosis factor (TNF-α), and other related chemokines and escalating the production of the anti-inflammatory cytokines like TGF-β, IL-4, and IL-10 (34, 35). Vitamin D also modulates the proinflammatory response by downregulating the TH1 and TH17 response and regulates the excessive inflammatory response during active mycobacterial infection. Nevertheless, vitamin D has suppressive role against proliferation of activated B cells, generation of plasma cells, and class switched memory cells, thereby reducing the levels of secreted immunoglobulin, which indirectly contributes to the downregulation of proinflammatory response and in turn promotes anti-inflammatory response by stimulating B-regs (36, 37). Vitamin D also modulates the inflammatory process by attenuating the expression of *M.tb* induced matrix metalloproteinase (MMPs) such as MMP-7, MMP-9, and MMP-10. With the help of CYP27B1 and Vitamin D receptors, Vitamin D3 induces cathelicidin gene encoded antimicrobial peptide LL37, which in turn increases the intracellular calcium levels and results in direct killing of *M.tb*. Increased levels of LL37 results in augmented differentiation of macrophages, bacterial killing through increased intracellular Ca2+ levels, and decreased *M.tb*-induced MMP 7, 9, and 10 and vitamin D-mediated TLR2 and 4 expression together with upregulation of IL-10. All these roles of Vitamin D have a definite impact on the progression and outcome of TB disease in an individual. Two meta-analyses have demonstrated that low serum vitamin D levels are associated with susceptibility to *M.tb* infection as well as progression to TB disease rather than low vitamin D levels being a consequence of the disease (38, 39). A study from South Africa has also identified that polymorphisms in gene associated with innate immunity, in combination with low vitamin D levels increase a child’s risk of developing TB disease or death (40).

Supplementation with vitamin D along with anti-TB treatment may be beneficial with respect to minimizing the excessive tissue damage that occurs during the active stage of TB disease. Several clinical trials have evaluated vitamin D supplementation as an adjunct therapy in the treatment for TB (41–44). However, results are conflicting, owing to variations in dose regimens and outcomes. Further investigations are needed to find the optimal concentration of vitamin D for supplementation with standard anti-TB drugs to optimize treatment, which could help to effectively manage both drug-sensitive and drug-resistant TB.

#### Metals

Essentially, metals like iron, manganese, copper, and zinc are very much needed for the survival of *M.tb* inside the macrophages. *M.tb* has accomplished features to acquire these metals from the cellular milieu and utilize them for survival and replication within the macrophages. Host immune system tackles the mycobacterial infection by sequestering metals like copper and zinc in the phagosomes beyond their normal levels and tries to kill them by metal poisoning. Alternatively, by means of nutritional immunity and with the help of translocation of metal transporter proteins, essential metals like iron and manganese will be depleted within the macrophages and thus prevent the replication of the bacilli (45, 46). In addition, iron homeostasis also differs between early and delayed TB-progressors, with higher ferritin and hepcidin concentrations observed among early TB-progressors among household contacts (47). It has been shown that both iron deficiency and overload exists in TB patients that affect the disease progression and clinical outcomes (48). Besides iron, manganese will also be depleted from the phagolysosomes through NRAMP1 with coordinated response of transferrin, ferritin, and hepcidin. Thus, the negative regulatory effect of iron and IFN-γ increases the antimicrobial function through increased production of IL-4/IL-10/IL-5/FOXP3, which results in decreased DCs maturation, function, and differentiation. In addition, pathways related to the production of Nos2 and TNFα mediate killing of *M.tb*. Innate immune response against *M.tb* can be strengthened by pumping of copper and zinc ions inside the phagolysosomes for bactericidal activity.

#### Minerals

In natural conditions, upon ingestion of immunogenic foreign bodies, the macrophages get stimulated and their intracellular Ca^2+^ ion concentration increases 10-fold than that of their non-stimulated state. The Ca^2+^ ions are very essential for several events associated with phagocytosis including activation of the Ca2+/CAM-dependent protein kinase II (CAMKII) and generation of reactive oxygen molecules. Selective inhibition of formation of Ca2+ CAM complex formation (pre translocation) or inhibition of CAMKII pathway on the surface of the phagosome (post-translocation) prevent the maturation of the phagosome (49). It is also been established that the effect of Ca2+/CAM/CAMKII complex on phagosome maturation is associated with series of events that are specifically involved in the phagosome–lysosome fusion (50). Table 1 gives an overview on the effect of macro and micro nutrient on immunomodulation that are discussed and referenced in this review.

**Table 1:** Role of macro- and micro-nutrients over immunomodulation during TB.

Nutrient deficiencies	Impact of the deficiency on immunomodulation	Reference
**Macro-nutrients**		
Proteins		
Total protein	↓ CD4/CD8 ratio	(14–16)
	↓ Expression of CD 25 and CD 27	
	↓ Production of IL-2 and IFN-γ	
Albumin	↓ Associated with death due to TB	(17)
Lipids		
n6PUFA	↓ Actin filament assembly and phagosome maturation *in vitro*	(19)
n3PUFA	↑ Actin filament assembly and phagosome maturation *in vitro*	(19)
n6 and n3 PUFA	Contrasting observation in animal model	(20)
Eicosanoids	Differentially regulated by *Mycobacterium tuberculosis* (*M.tb)* to avoid apoptosis	(21)
Sphingosine-1-phosphate (S1P)	*M.tb* dephosphorylate S1P and thereby prevents phagosome maturation	(22)
**Micro-nutrients**		
Vitamins		
Vitamin A	↓ Cell-mediated responses and lympho-proliferative responses	(24, 25)
Vitamin B6	↓ Lymphocyte and natural killer cell activities	(26, 27)
Vitamin C	↑Reactive oxygen species and tissue injury due to inflammation caused by *M.tb*	(28)
Vitamin E	↑Oxidative stress and suppressed T-cell function	(29, 30)
Vitamin D	↓ Macrophage differentiation and phagocytosis	(31–39)
	↓ Levels of cathelicidins, β-defensin, hepcidin antibacterial protein, and hCAP18	
	↑ Proinflamatory cytokines and ↓ anti-inflammatory cytokines	
	↑ MMP 7, 9, and 10	
Metals		
Copper and zinc	Decreased killing of mycobacteria in phagosomes	(45–48)
Iron and manganese	Overload of TB bacilli affects TB diseases progression and clinical outcome	(45–48)
	NOS2 and TNFα pathways affected	
Minerals		
Ca2+	Decreased phagocytosis	(49, 50)
	Generation of reactive oxygen molecules	
	Phagosome lysosome fusion	

**Figure 1:**
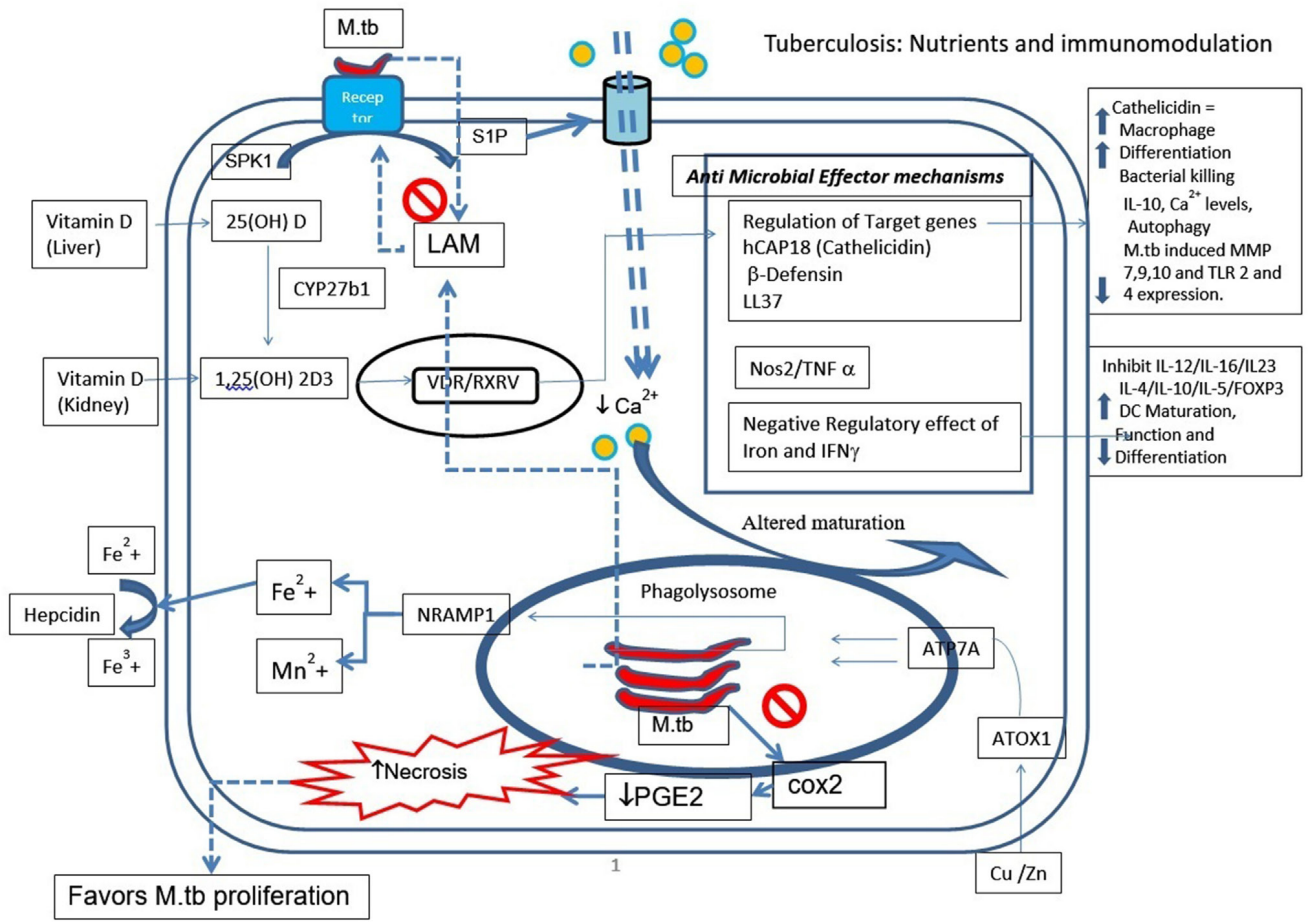
Tuberculosis: nutrients and immunomodulation.

In brief, Figure 1 represents a schematic diagram of the role of all nutrients explained above during *M.tb* infection. Upon infection, a series of antimicrobial effector mechanisms will be triggered by macrophages. *M.tb* also inhibits cycoloxygenase2 (cox2) pathway and limits prostaglandin 2 (PGE2), which is shown to be involved in membrane repair and apoptosis. By limiting PGE2, *M.tb* diverts the apoptotic pathway toward necrosis, which is essential for *M.tb* survival and proliferation.

### Body Mass Index (BMI) and Regulatory Cytokines of TB

Body Mass Index is a reliable estimate of the nutritional status of an individual. An inverse log-linear relationship has been shown between BMI and the incidence of TB across different settings of TB burden—a 14% reduction in the incidence of TB per unit increase of BMI (51). The biological mechanism linking BMI with risk of TB remains an enigma. Cytokines of the innate and adaptive immune systems play an important role in mediating the immune response to *M.tb*. A recent study has shown a link between BMI and inflammatory cytokines of TB. Individuals with low BMI have diminished circulating levels of proinflammatory (IFN-γ, TNF-α, IL-22, IL-1α, IL-1β, and IL-6) cytokines but elevated levels of regulatory cytokines (IL-10, TGF-β, IL-5, IL-13) (52). This group demonstrated a positive correlation between the circulating levels of pro-inflammatory cytokines and high BMI (between 25 and 29.9) and a negative correlation between the circulating levels of anti-inflammatory cytokines and low BMI (53, 54). These data suggest a protective mechanism of BMI against progression of TB infection to disease by altering the cytokine milieu of an individual.

### Boosting Immunity through Nutritional/Food Supplementation

The above evidences clearly establish the fact that chronic nutritional deficiency (under nutrition or malnutrition) compromises the innate and adaptive immunity of an individual, leading to immunodeficiency, which increases one’s susceptibility to disease contributing to increased morbidity and mortality (55). Number of clinical studies, trials, and meta-analysis of nutritional or food supplementation among TB patients have shown varied results in various settings. In general, it is known that supplementation of micro-/macro-nutrients to TB patients not only improves the body weight and BMI but also has an effect on T-cell function, sputum conversion, relapse, physical activity, and mortality (26, 28, 48, 56–60). Studies have also shown the effects of nutrient supplementation on the pharmacokinetics of the ATT drugs, which helps in improving the treatment outcome by increasing the bioavailability of the anti-TB drugs (61, 62). However, there is no documented evidence that any specific food on its own or a specific quantity can alter the course of TB disease or can for that matter be effective in the treatment of malnutrition.

### Future Research

Though the relationship between nutritional status and immunology is very evident, many questions remain unanswered and needs further research to establish the clinical utility of this relationship. Few of the questions that can be answered through operational research in the field settings include

1. The ideal nutritional supplementation for the prevention and management of infectious diseases
2. Better understanding of interactions between immune signaling pathways and resistance to diseases
3. Advanced nutriogenomics studies for predisposition of the infectious diseases—in terms of transcriptomics, proteomics, and metabolomics with respect to dietary signals
4. To study the role of diet and lifestyle and how it affects the health
5. Role of nutrition among elderly and pediatric patient population to boost immunity
6. Effect of lipids over inflammation and infection, especially in TB and emergence of drug resistance
7. Association of impaired immune response and poor dietary intake
8. Association of TB infection and disease with Hidden hunger (micronutrient deficiency).

## Conclusion

In general, poor nutrition leads to macro- and micro nutrient deficiencies, which can lead to immunodeficiency, both cell mediated and humoral immune response. This secondary immunodeficiency, in turn, affects one’s ability to fight infection resulting in increased susceptibility to diseases including TB. There seems to be a definite role for nutritional or food supplementation in alleviating the detrimental effects produced by malnutrition on immune response to TB. However, there is no documented evidence that any specific food on its own or a specific quantity can alter the course of TB disease or can for that matter be effective in the treatment of malnutrition. There is experimental evidence that some nutrients may be helpful but further clinical studies and randomized clinical trials will be needed for them to be recommended and to better understand the link between malnutrition, TB and impaired immunity.

## Author Contributions

PC conceived the idea, collected data, wrote, and edited the manuscript. SN and RB contributed for writing the manuscript. ST edited the manuscript.

## Conflict of Interest Statement

The authors declare that the research was conducted in the absence of any commercial or financial relationships that could be construed as a potential conflict of interest.
